# Pangenome Evidence for Higher Codon Usage Bias and Stronger Translational Selection in Core Genes of *Escherichia coli*

**DOI:** 10.3389/fmicb.2016.01180

**Published:** 2016-08-03

**Authors:** Shixiang Sun, Jingfa Xiao, Huiyong Zhang, Zhang Zhang

**Affiliations:** ^1^CAS Key Laboratory of Genome Sciences and Information, Beijing Institute of Genomics, Chinese Academy of SciencesBeijing, China; ^2^BIG Data Center, Beijing Institute of Genomics, Chinese Academy of SciencesBeijing, China; ^3^University of Chinese Academy of SciencesBeijing, China; ^4^College of Life Sciences, Henan Agricultural UniversityZhengzhou, China

**Keywords:** pangenome, codon usage bias, translational selection, mutation, core genes, strain-specific genes

## Abstract

Codon usage bias, as a combined interplay from mutation and selection, has been intensively studied in *Escherichia coli*. However, codon usage analysis in an *E. coli* pangenome remains unexplored and the relative importance of mutation and selection acting on core genes and strain-specific genes is unknown. Here we perform comprehensive codon usage analyses based on a collection of multiple complete genome sequences of *E. coli*. Our results show that core genes that are present in all strains have higher codon usage bias than strain-specific genes that are unique to single strains. We further explore the forces in influencing codon usage and investigate the difference of the major force between core and strain-specific genes. Our results demonstrate that although mutation may exert genome-wide influences on codon usage acting similarly in different gene sets, selection dominates as an important force to shape biased codon usage as genes are present in an increased number of strains. Together, our results provide important insights for better understanding genome plasticity and complexity as well as evolutionary mechanisms behind codon usage bias.

## Introduction

As an important organism in biotechnology and microbiology, the completion of whole genome sequencing of *Escherichia coli* accomplished in 1997 (Blattner et al., 1997) has laid a significant foundation for fully studying its genome (Zimmer, 2009; Lukjancenko et al., 2010). Since then, many studies performed analyses on *E. coli* at different aspects for characterizing its genome diversity (Rasko et al., 2008; Touchon et al., 2009; Lukjancenko et al., 2010), horizontal gene transfer (Jain et al., 1999; Ochman et al., 2000; Gogarten and Townsend, 2005), pathogenicity (Kaper et al., 2004; Croxen and Finlay, 2010), and evolutionary process (Clermont et al., 2000; Elena and Lenski, 2003; Lewis et al., 2010). Among them, codon usage studies have been extensively conducted in *E. coli*, demonstrating heterogeneity in synonymous codon usage and revealing that codon usage bias principally arises from a complex interplay between mutation and selection (Bulmer, 1991; Sharp et al., 1993; dos Reis et al., 2003; Hershberg and Petrov, 2008; Plotkin and Kudla, 2011).

Initially, studies on *E. coli* have identified selection as a major force since codon usage in highly expressed genes is positively correlated with tRNA abundance (Ikemura, 1981, 1985; Gouy and Gautier, 1982). Subsequently, evidence has further accumulated that mutation is also an important driving force shaping heterogeneous codon usage in a variety of bacteria, including *E. coli* (Sueoka, 1988; Knight et al., 2001; Chen et al., 2004). Meanwhile, it has been argued that mutation alone cannot lead to nonrandom nucleotide composition in many bacteria species (Hershberg and Petrov, 2010; Hildebrand et al., 2010) and selection may play an important role in driving GC content variation (Stoletzki and Eyre-Walker, 2007; Sharp et al., 2010; Raghavan et al., 2012). Recent studies have shown that another confounding factor, namely, GC-biased gene conversion, which is believed to be independent from selection, may provoke the nonrandomness of base composition and the heterogeneity of synonymous codon usage in *E. coli* as well as other bacteria (Touchon et al., 2009; Lassalle et al., 2015; Reichenberger et al., 2015). Although codon usage has been extensively studied in *E. coli*, it can be seen that the relative importance of mutation and selection operating on codon usage has been still controversial (Knight et al., 2001; Stoletzki and Eyre-Walker, 2007; Ran et al., 2014) and previous studies performed codon usage analysis primarily on individual genomes (dos Reis et al., 2003).

The availability of complete genome sequences of multiple different strains for a given species, collectively constituting this species pangenome, offers a new strategy to fully capture bacterial genome plasticity and complexity and to unveil the underlying evolutionary mechanisms associated with a wide diversity of environments (Medini et al., 2005; Vernikos et al., 2015). As a pangenome is composed of core genes that are present in all strains, dispensable genes that are present in two or more strains, and strain-specific genes that are unique to single strains, genome sequences of multiple *E. coli* strains enable in-depth analyses on codon usage in a pangenome context. Recent studies conducted pangenome analysis based on multiple *E. coli* strains, primarily focusing on identification of core genome, and dispensable genome (Lukjancenko et al., 2010), comparison of commensal and pathogenic isolates (Rasko et al., 2008), and investigation of gene variation and phylogeny inference (Kaas et al., 2012). However, codon usage analysis in an *E. coli* pangenome remains unexplored and importantly, very little is known about the relative importance of mutation and selection acting on core genes and strain-specific genes. Toward this end, here we perform comprehensive codon usage analyses based on a collection of multiple complete genome sequences of *E. coli*, explore the major force in shaping biased codon usage in the *E. coli* pangenome, and investigate whether mutation and selection act differentially on synonymous codon usage between core genes and strain-specific genes.

## Materials and methods

### Data collection

We retrieved 61 complete genome sequences of *E. coli* from the National Center for Biotechnology Information (NCBI) (ftp://ftp.ncbi.nlm.nih.gov/genomes/all/), and summarized their details in Table S1. To reduce redundancy of these retrieved genomes, we selected the strains that are evolutionarily divergent based on the genomic blast dendrogram. As a result, a collection of 26 genome sequences was used for pangenome analysis (Figure S1). For each strain, horizontally transferred genes were identified by Islandviewer (Dhillon et al., 2015) and detailed information of horizontally transferred genes identified for all strains were summarized into Table S2. We obtained RNA-Seq data for 4 *E. coli* strains from SRA (http://www.ncbi.nlm.nih.gov/sra/; accession numbers: SRR1184439, SRR1183094, SRR1185100, and SRR915686). Reads were filtered using FASTX-Toolkit (http://hannonlab.cshl.edu/fastx_toolkit/index.html) and mapped to the reference genomes with Bowtie2 (Langmead and Salzberg, 2012). We downloaded tRNA copy number data for *E. coli* from GtRNAdb (Chan and Lowe, 2016).

### Pangenome analysis

Based on a total of 95,439 genes from 26 strains, we used OrthoMCL (Fischer et al., 2011) and PanGP (Zhao et al., 2014) for pangenome analyses. As a result, we clustered all genes into 6797 clusters and further grouped the *E. coli* pangenome into five gene sets: strain-specific genes (that are present in only one strain; *n* = 1812 in 1723 clusters), lowly-shared genes (that are shared between 2 and 9 strains; *n* = 6728 in 1467 clusters), moderately-shared genes (that are shared found between 10 and 17 strains; *n* = 5776 in 398 clusters), highly-shared genes (that are shared between 18 and 25 strains; *n* = 24,697 in 1041 clusters), and core genes (that are present in all 26 strains; *n* = 59,426 in 2168 clusters, Table S3). However, considering that genomes may have paralogs (e.g., 59,426 vs. 26 × 2168 = 56,368 in core genes), therefore, for each cluster, we defined representative genes as genes after removal of paralogs. Analyzed results thereinafter were based on all genes, while those based on representative genes that lead to consistent conclusions were presented as Supplementary Materials.

### Codon usage analysis

To avoid artifacts caused by methodology, we adopted multiple different measures for estimating codon usage bias, including CDC (Codon Deviation Coefficient; Zhang et al., 2012), CAI (Codon Adaptation Index; Sharp and Li, 1987), *Nc* (Effective Number of Codons; Wright, 1990), and *Nc*′ (a variant of *Nc*) (Novembre, 2002). It is noted that CAI and CDC produce values varying from 0 (no bias) to 1 (maximum bias), whereas *Nc* and *Nc*′ range from 20 (maximum bias) to 61 (no bias). To investigate the variation trend of codon usage bias across different gene sets and examine whether different measures present consistent trends, therefore, we rescaled *Nc* and *Nc*′ to make them range from 0 (no bias) to 1 (maximum bias) by using the formula (61–*X*)/41, where *X* = *Nc* or *Nc*′. To avoid stochastic errors, genes that are shorter than 100 codons were excluded from this analysis, as codon usage estimation might be biased in shorter genes (Kessler and Dean, 2014). In neutrality-plot, GC_12_ is the mean of GC contents averaged over the first two codon positions (viz., GC_1_ and GC_2_, respectively). The cosine similarity metric was used to estimate the degree of similarity between tRNA abundance and relative synonymous codon usage (RSCU) and formulated as below,

(1) cosθ=∑k=1nXkYk∑k = 1nXk2∑k = 1nYk2,

where *n* is the total number of the codons and for a given codon *k, X*_*k*_ is tRNA copy number and *Y*_*k*_ is the RSCU value. The cosine similarity metric ranges from 0 (completely different) to 1 (identical).

### Correspondence analysis (COA)

As a useful statistical method to analyze the deviation of the RSCU value, COA provides a major trend of factors related to codon usage in different gene sets. In COA, genes were plotted into a 59-dimensional hyperspace according to the usage of the 59 informative codons, excluding AUG, UAA, UAG, UGA, and UGG. Generally, if the variability of one axis is >10%, this axis indicates a major variation trend (Greenacre, 1984).

### ENC-plot

It is an effective way to explore heterogeneity in codon usage by plotting *Nc* values against GC_3_ (Wright, 1990). In ENC-plot, *estimated Nc* was obtained by ENCprime (Novembre, 2002) and *expected Nc* was calculated using the formula 2 + *X* + 29/[*X*^2^ + (1−*X*)^2^], where *X* = GC_3_. In general, if a gene is under strong mutation rather than selection, *estimated Nc* will be close to *expected Nc*, with no or slight deviation. Otherwise, a large deviation between *estimated Nc* and *expected Nc* indicates strong selection in influencing this gene's codon usage.

### Statistical analysis

All statistical tests were carried out using the statistical analysis software SPSS. The differences in CDC, CAI, *Nc, Nc*′, nucleotide compositions, and similarity between tRNA abundance and RSCU were analyzed by one-way ANOVA. Spearman's rank correlation analysis was used in COA and expression level correlation.

## Results and discussion

**Figure 1:**
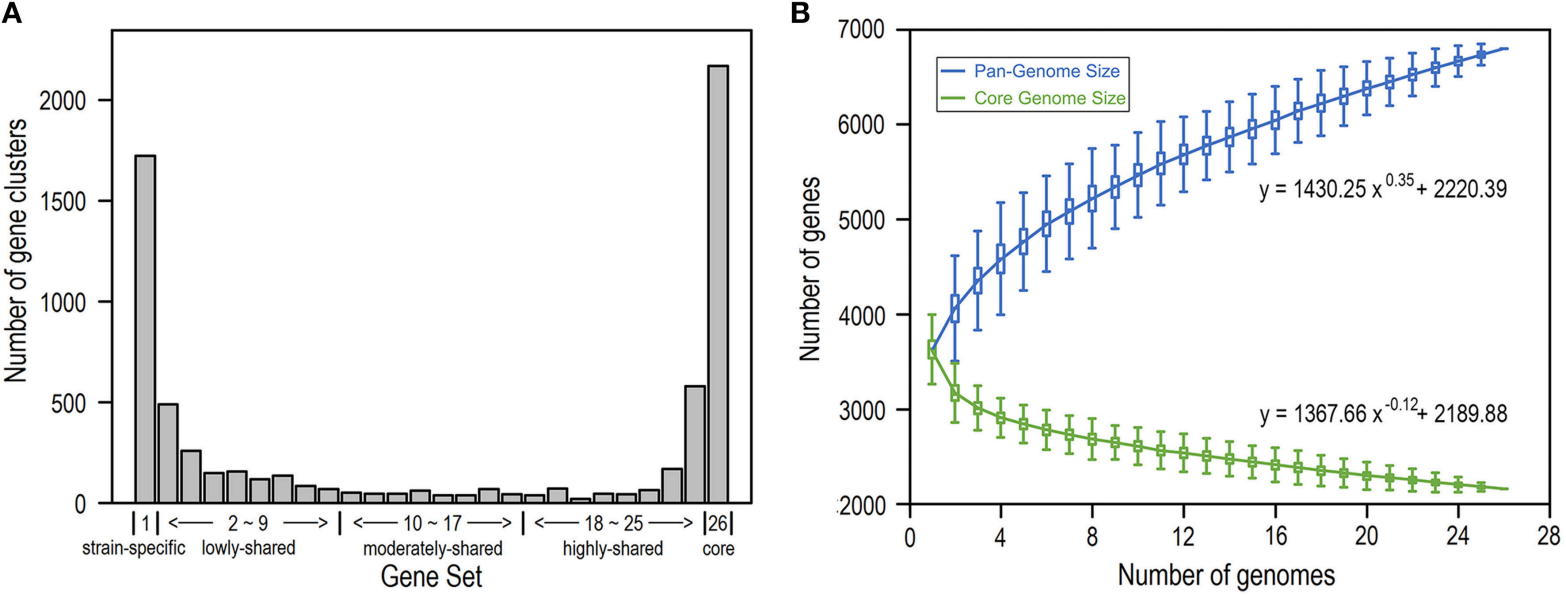
*****E. coli*** pangenome and core genes based on 26 isolates. (A)** Number of gene clusters shared in 1–26 isolates, respectively. According to their presence in different number of isolates, genes were further grouped into five gene sets: strain-specific genes, lowly-shared genes, moderately-shared genes, highly-shared genes, and core genes. **(B)** Pangenome size and core-genome size when the number of isolates varies from 1 to 26.

We build the *E. coli* pangenome based on a collection of complete genome sequences from 26 closely divergent isolates (Table S1). Considering the possibility of acquisition of genes by horizontal transfer, which consequently may lead to higher heterogeneity in codon usage as well as nucleotide composition (Koonin et al., 2001), we perform pangenome analysis for all *E. coli* genes by removal of horizontally transferred genes (Table S2) and identify genes that are present in 1–26 isolates, respectively (Figure 1; see Section Materials and Methods). As a result, we obtain 2168 core gene clusters (that are present in all 26 isolates) and 1723 strain-specific gene clusters (that are present in only one isolate) (Figure 1A). Noticeably, core genes and strain-specific genes are relatively abundant, presumably indicating that *E. coli* is not only conservative in core functions but also is active in gene birth for adaptation to new environments (Davids and Zhang, 2008). When more isolates are included, the *E. coli* pangenome becomes larger and the size of core genes decreases dramatically to be smooth at larger number of isolates (Figure 1B), indicating that *E. coli* is an open genome (Tettelin et al., 2008; Lukjancenko et al., 2010).

### GC content and gene length in the *E. coli* pangenome

**Figure 2:**
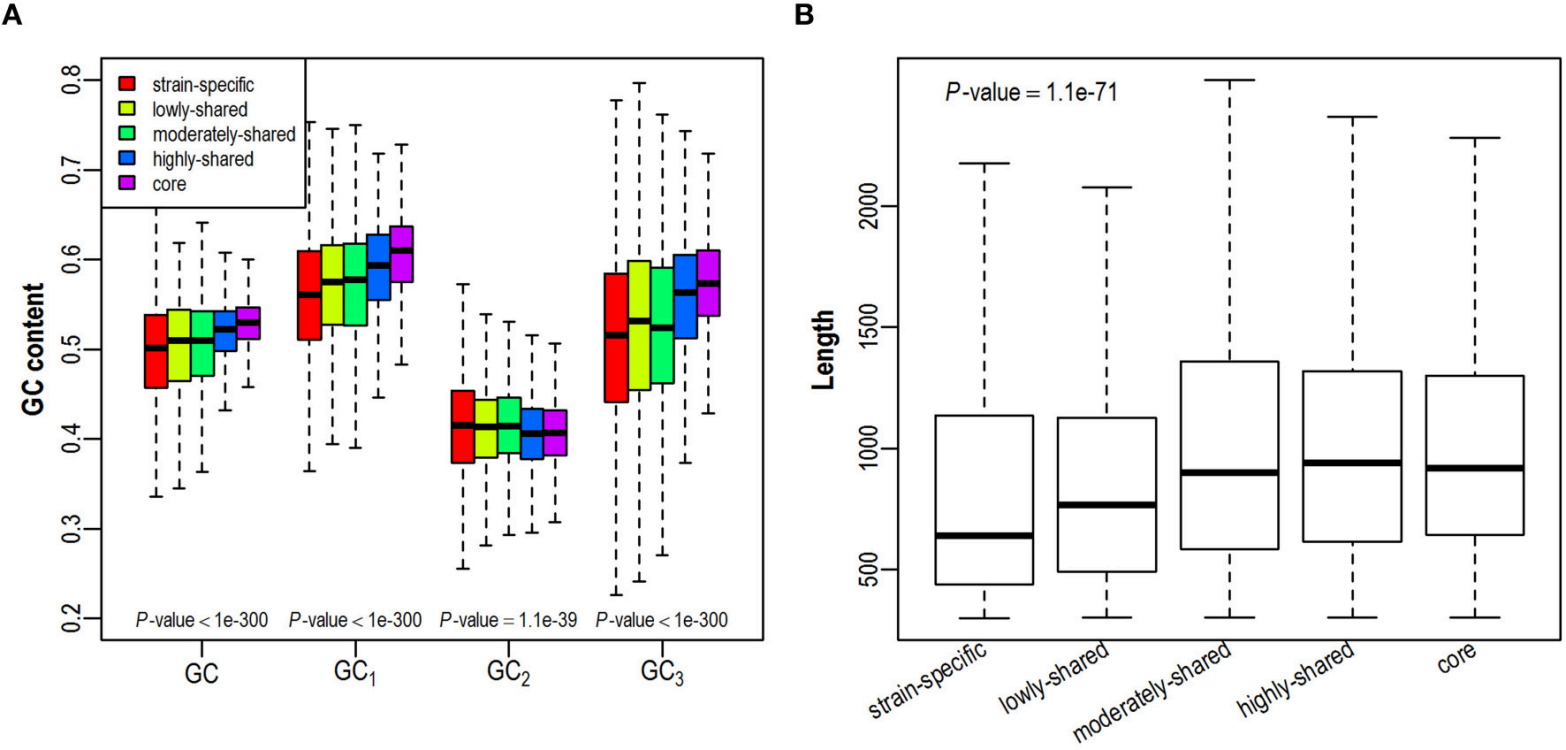
**Distribution of GC contents (A) and gene length (B) across five gene sets in the ***E. coli*** pangenome**. GC contents at three codon positions are denoted as GC_1_, GC_2_, GC_3_, respectively.

As GC content is highly related to synonymous codon usage, we first investigate whether a gene's GC content is dependent on its presence in different number of isolates (Figure 2A and Figure S2A). We find that GC content is higher in core genes and lower in strain-specific genes, exhibiting a positive correlation with gene presence. As GC contents at three different codon positions (denoted as GC_1_, GC_2_, GC_3_, respectively) correlate closely yet differentially with the overall GC content (Hu et al., 2007), we further investigate the trend of positional GC contents across different gene sets. Intriguingly, GC_1_ and GC_3_ correlate positively with gene presence in the pangenome, presenting comparable trends as GC content does. On the contrary, GC_2_ is relatively constant across all examined gene sets, probably due to stronger selection at this position since any substitution in the second codon position leads to the amino acid replacement and protein structure variation (Gu et al., 2004). As previous studies have shown that gene length is positively correlated with GC content (Oliver and Marin, 1996; Li and Du, 2014), we further examine the variation of gene length in all five gene sets. Consistently, core genes tend to be longer than strain-specific genes (Figure 2B and Figure S2B). Taken together, with an increased presence in more isolates, genes tend to have higher GC contents and longer sequences.

### Codon usage bias (CUB) and translational selection

**Figure 3:**
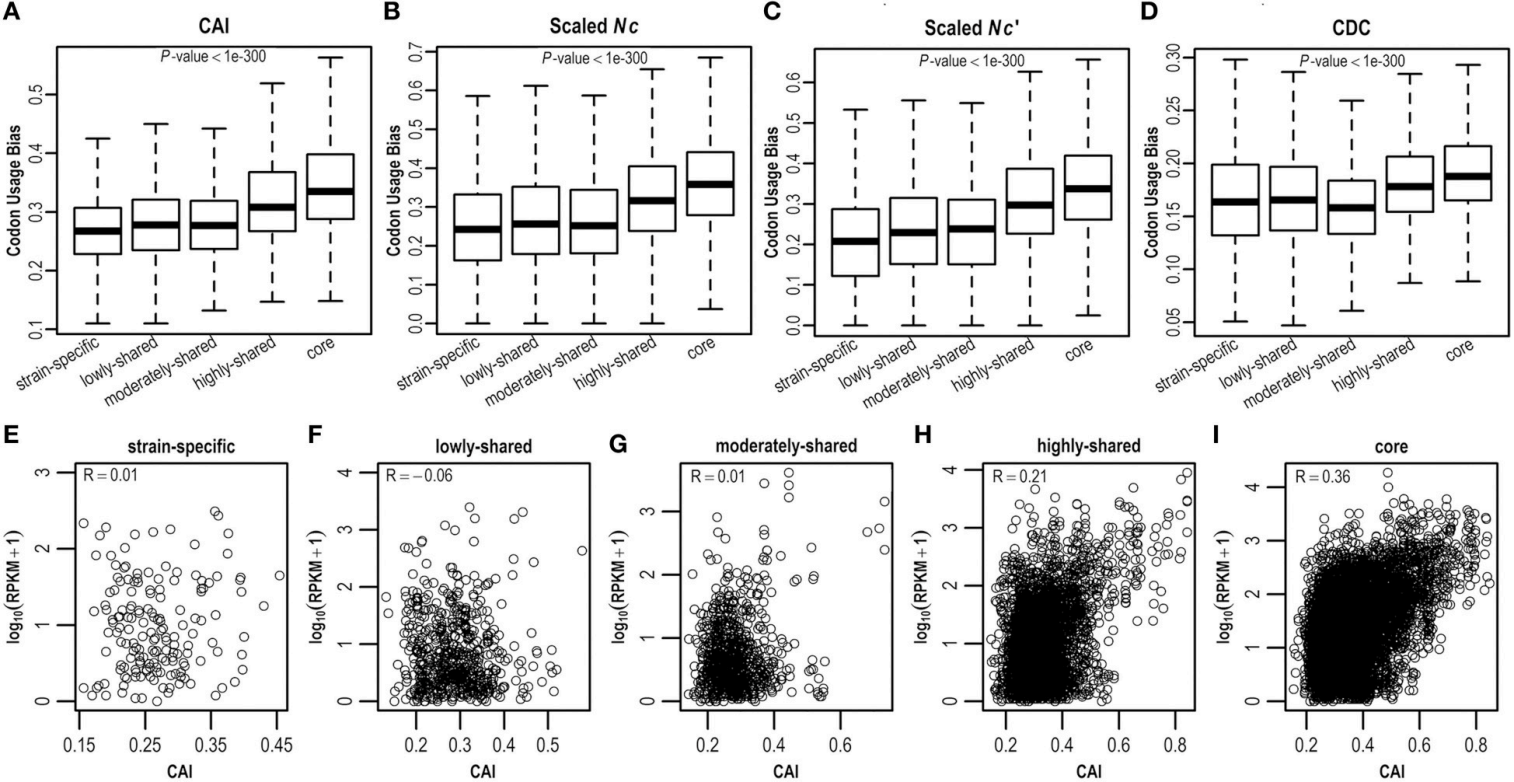
**Codon usage bias in the ***E. coli*** pangenome estimated by four different measures, viz., (A) CDC, (B) CAI, (C) ***Nc***, and (D) ***Nc***′**. Correlation between CAI and gene expression level was examined in **(E)** strain-specific genes, **(F)** lowly-shared genes, **(G)** moderately-shared genes, **(H)** highly-shared genes, and **(I)** core genes, respectively.

As *E. coli* genes have different evolutionary histories and accordingly may have experienced differential forces from mutation and selection shaping synonymous codon usage, here we estimate CUBs for *E. coli* genes in the context of pangenome. Clearly, core genes possess more biased codon usage than strain-specific genes (*P* < 0.05; Figures 3A–D and Figure S3). Specifically, core genes have highest CUBs, followed by highly-shared, moderately-shared, and lowly-shared genes, whereas strain-specific genes present lowest CUBs. This result is consistently observed by different CUB measures (Figures 3A–D and Figure S3), although they adopt different strategies for CUB estimation. To further examine what genes possess higher CUBs in different gene sets, we sort genes in term of CUB and find that the top 10 in core genes are most ribosomal proteins (that are believed to be highly expressed), whereas the top 10 in strain-specific genes are almost hypothetical proteins (Table S4).

As biased codon usage is thought to arise from selection for translational efficiency and/or accuracy, it is believed that a positive correlation between CUB and gene expression level is indicative of translational selection (Ikemura, 1981; Plotkin and Kudla, 2011; Ma et al., 2014). To decipher whether translational selection is also associated with gene presence in the context of a pangenome, we collect RNA-Seq data (Figure S4) for *E. coli* and examine the correlation between CUB and gene expression level in five different gene sets where genes are shared in different numbers of isolates (Table S5). As a result, we find that the correlation between CUB and gene expression level is positively stronger in core genes by comparison with strain-specific genes, indicating that translational selection acts stronger in core genes (Figures 3E–I and Figure S5).

**Figure 4:**
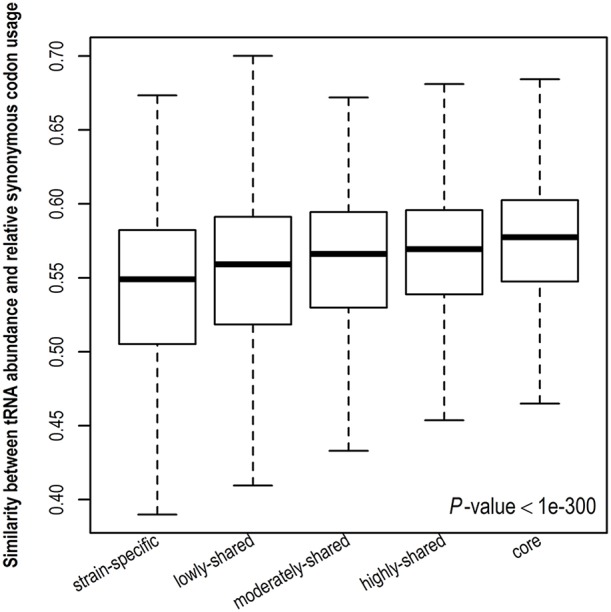
**Similarity between tRNA abundance and relative synonymous codon usage**. The cosine similarity metric was used, indicating the degree of similarity between tRNA abundance and relative synonymous codon usage and ranging from 0 (completely different) to 1 (identical).

Stronger translational selection indicates that synonymous codon usage is more biased toward tRNA abundance. To further validate the result derived from gene expression level, we examine the similarity between tRNA abundance and RSCU among different gene sets (Figure 4 and Figure S6). A higher similarity suggests close correspondence between codon usage and tRNA abundance. Consistently, we observe that the similarity between tRNA abundance and RSCU is positively correlated with gene presence and core genes take the highest similarity, indicating that codon usage in core genes is more biased toward tRNA abundance, viz., core genes are under the strongest translational selection among five gene sets. Taken together, in contrast to other genes, core genes tend to have higher CUBs and experience stronger translational selection.

### Heterogeneity of mutation and selection acting on core and strain-specific genes

To dissect factors influencing codon usage in different *E. coli* genes, we first perform correspondence analysis on RSCU across all five different gene sets (Figure S7). Considering that the first principal axis can explain the majority of codon usage (>10%), we then analyze the correlation between the first axis and 65 factors for each gene set (Table S6). We find that there is a significant correlation between GC_3_ and the first axis in strain-specific genes (*R* = 0.92, *P* < 1e-300), but its absolute value drops gradually as genes are present in more isolates (0.92 in lowly-shared genes, 0.91 in moderately-shared genes, and 0.61 in highly-shared genes) and is significantly lower in core genes (*R* = 0.28, *P* < 1e-300). On the other hand, CAI presents opposite trends that core genes have the highest significant correlation (*R* = 0.94, *P* < 1e-300) and strain-specific genes have the lowest correlation (*R* = 0.63, *P* = 3.3e-201). Collectively, these results demonstrate that selection in connection with expression level indicated by CAI dominates core genes, whereas mutation reflected by GC_3_ dominates strain-specific genes. However, it should be noted that mutation is a genome-wide force in shaping synonymous codon usage (Chen et al., 2004) and accordingly may act similarly in each gene set. In spite of this, our results clearly show that core genes are under stronger selection than strain-specific genes, indicating that factors influencing codon usage variation are heterogeneous in different gene sets.

**Figure 5:**
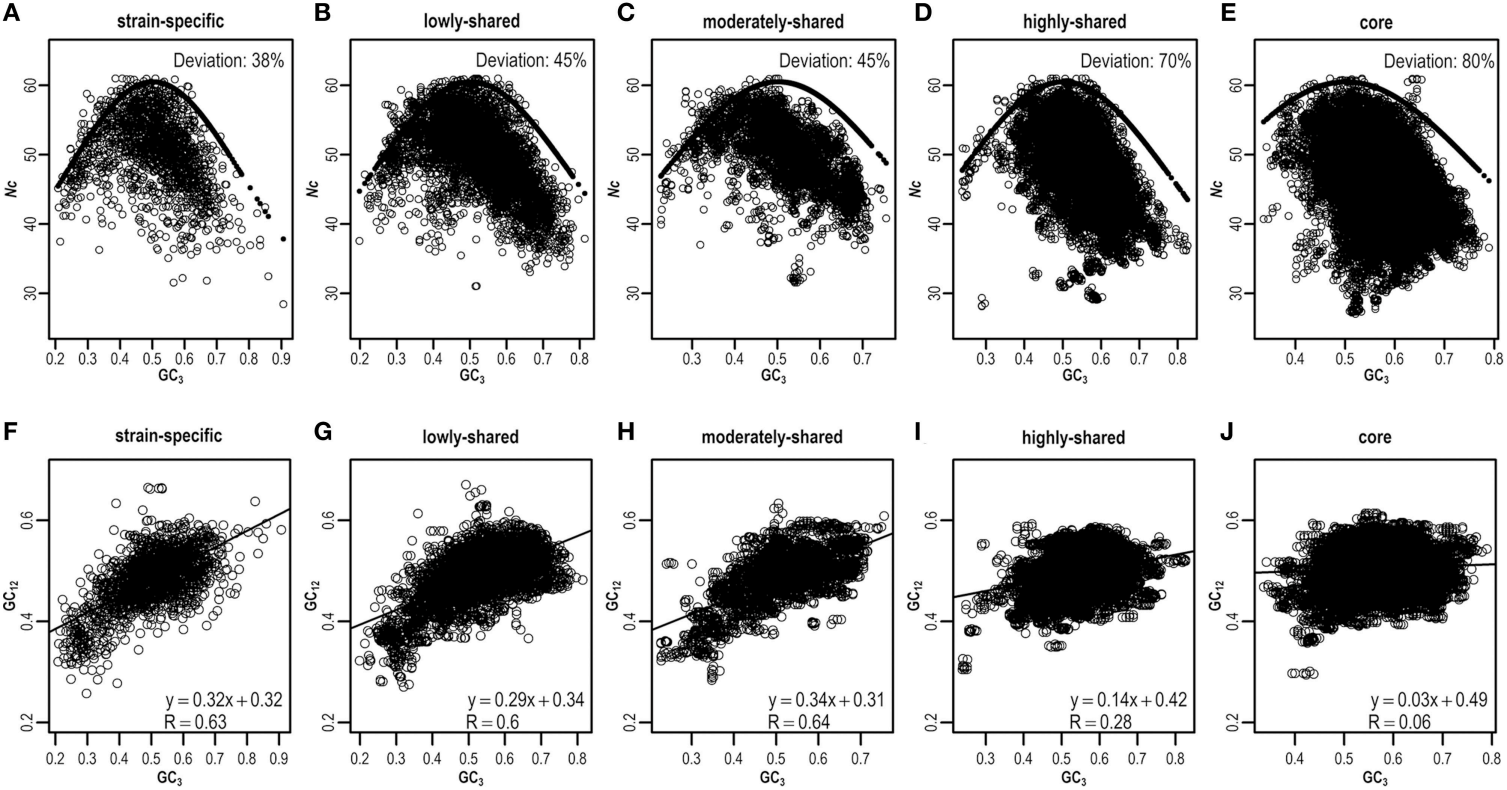
**ENC-plot (A–E) and neutrality-plot (F–J) across different gene sets**. The expected and estimated values in ENC-plot are indicated by solid and hollow circles, respectively. For any given gene, if its deviation value, calculated by *(expected–estimated)/expected*, is greater than a threshold (default = 0.15), this gene is assumed to deviate from the expected ENC curve. Similar results can be found in Table S7 when considering different thresholds.

As ENC-plot is widely used to investigate the influence of mutation and selection acting on codon usage (Wright, 1990), we plot *Nc* against GC_3_ (Figures 5A–E and Figures S8A–E) to identify the main factor in shaping heterogeneous codon usage in the pangenome context. Agreeing with results presented above, most strain-specific genes are around the expected ENC curve, indicating that these genes are driven primarily by mutation (Figure 5A), whereas core genes are deviated from the expected curve, suggesting that selection is a major force operating on core genes (Figure 5E). Quantitatively, we estimate the percentage of genes that are deviated from the expected curve and clearly find the core genes present higher deviations than strain-specific genes (Figures 5A–E and Table S7). These results suggest that although mutation exerts genome-wide influences on codon usage (Chen et al., 2004), selection dominates as an important factor to influence codon usage in core genes.

To further validate the result derived from ENC-plot, we also perform neutrality-plot that is based on nucleotide contents to quantify the relative ratio between mutation and selection (Sueoka, 1988). Based on the *E. coli* pangenome, we hypothesize that nucleotide content at the third codon position is different from that at the first two codon positions, which is expected to be more pronounced in core genes than strain-specific genes. To test this hypothesis, we conduct neutrality-plot in different gene sets (Figures 5F–J and Figures S8F–J). We find that the correlation between GC_3_ and GC_12_ (mean value of GC_1_ and GC_2_) is significantly positive in strain-specific genes (*R* = 0.63, *P* = 3.6e-204; Figure 5F), but drops gradually in highly-shared, moderately-shared, and lowly-shared gene sets (Figures 5G–I), and becomes very weak or nearly absent in core genes (*R* = 0.06, *P* = 3.1e-42; Figure 5J). These results show that strain-specific genes have smaller differences in nucleotide composition between GC_3_ and GC_12_, whereas core genes have the larger difference. In addition, the slope of GC_3_–GC_12_ regression function decreases from 0.32 in strain-specific genes to 0.03 in core genes. It should be noted that the slope equals to 0 represents no effect of directional mutation pressure (complete selective constraints) and 1 stands for the complete neutrality (Sueoka, 1988).

Taken collectively, results derived from ENC-plot and neutrality-plot provide evidences that core genes are under stronger selection than strain-specific genes. Agreeing with previous studies that translational selection is found in *E. coli* (dos Reis et al., 2003), our results provide further detailed evidence from the pangenome level that stronger translational selection in *E. coli* is contributed considerably by core genes. Considering that core genes are majorly comprised by housekeeping genes (Bentley, 2009) and encode basic functions and phenotypical traits related to the basic biology of the species (Medini et al., 2005; Monk et al., 2013), stronger selection provides high translational accuracy to minimize the missense and nonsense errors (Stoletzki and Eyre-Walker, 2007; Hershberg and Petrov, 2008) and accelerates the translation elongation in protein expression (Ran et al., 2014), which is advantageous for genome stability in species evolution. As for strain-specific genes, mutation and weak selection combined contribute to formation of new genes, which increases the genome plasticity and species diversity, provides supplementary biochemical pathways (Medini et al., 2005) and acquires selective advantages (Mongodin et al., 2013) for certain strains that live in different circumstances. Therefore, our results provide important insights for better understanding genome plasticity and complexity as well as evolutionary mechanisms behind codon usage bias.

## Author contributions

SS analyzed the data and drafted the manuscript. JX and ZZ designed the research. HZ and ZZ revised the manuscript. All authors have approved the final version of the article. All authors agree to be accountable for all aspects of the work in ensuring that questions related to the accuracy or integrity of any part of the work are appropriately investigated and resolved.

## Funding

This work was supported by grants from National Programs for High Technology Research and Development (863 Program; 2014AA021503 and 2015AA020108) and the “100-Talent Program” of Chinese Academy of Sciences.

### Conflict of interest statement

The authors declare that the research was conducted in the absence of any commercial or financial relationships that could be construed as a potential conflict of interest.
