# Supplementary figures and images for: Pangenome Evidence for Higher Codon Usage Bias and Stronger Translational Selection in Core Genes of Escherichia coli
Source: Front Microbiol. 2016 Aug 3;7:1180. doi: 10.3389/fmicb.2016.01180 (PMC4971109; doi:10.3389/fmicb.2016.01180)

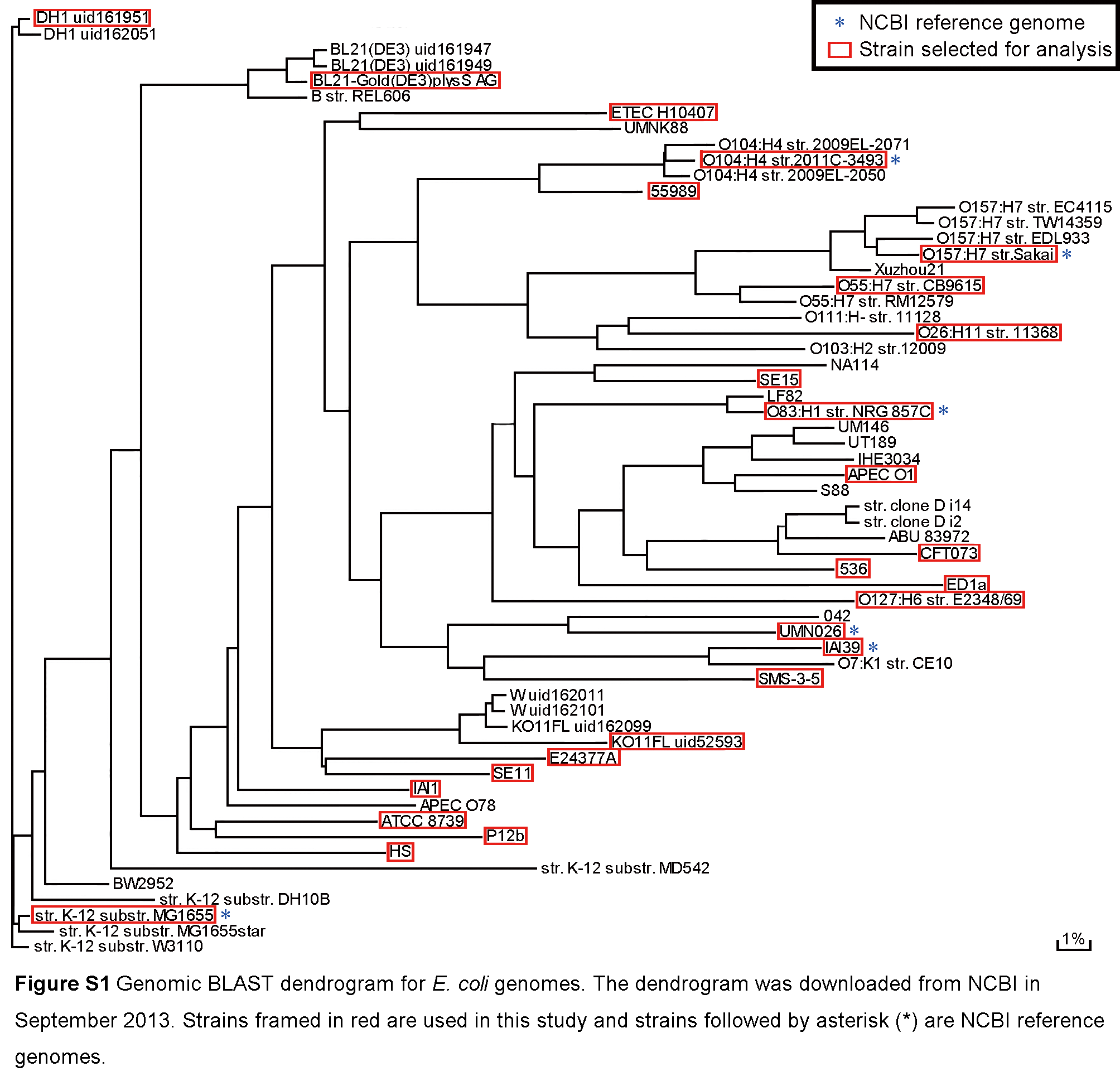

Supplement: Supplementary file 8 [file Image1.TIF]

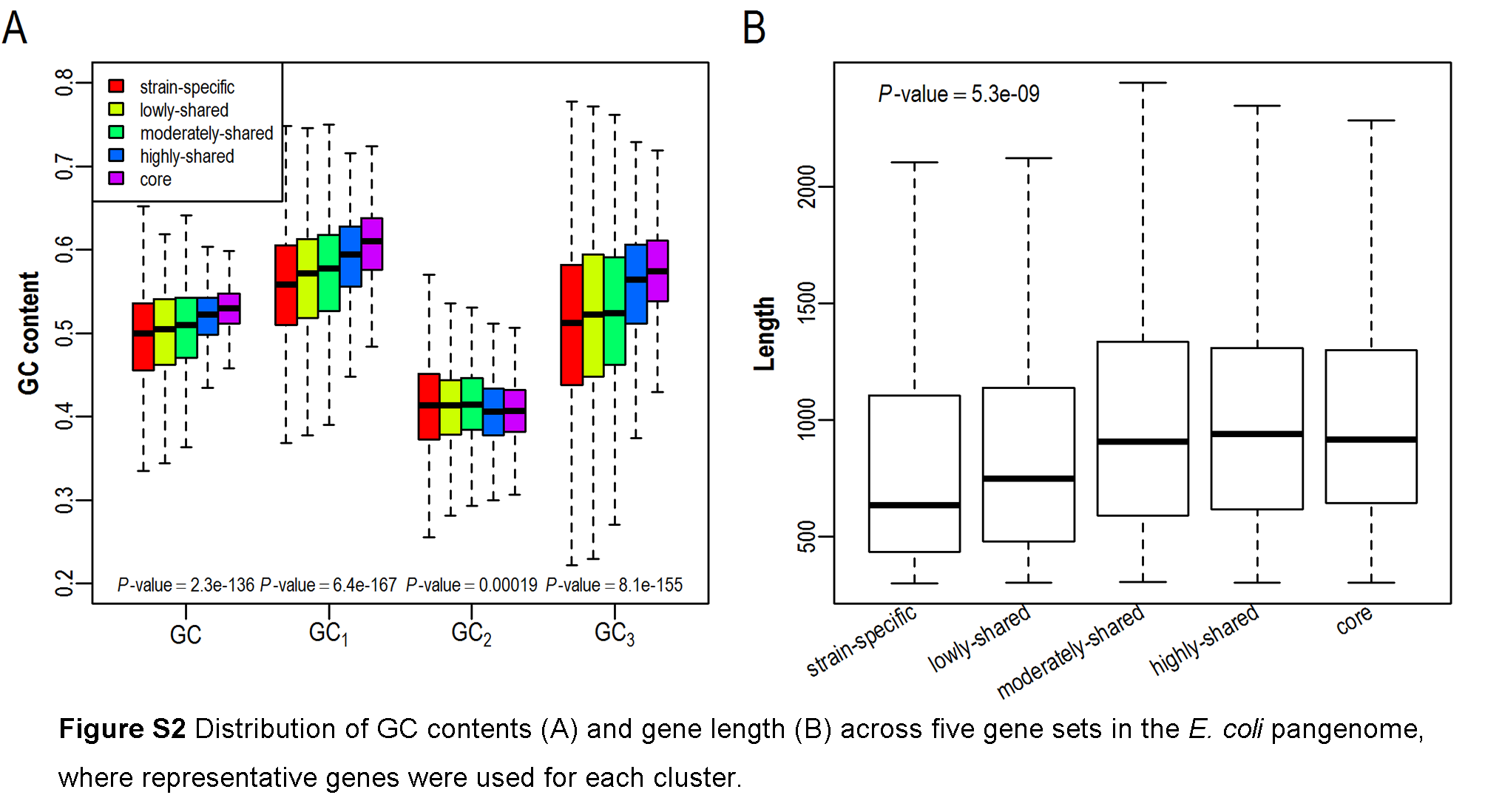

Supplement: Supplementary file 9 [file Image2.TIF]

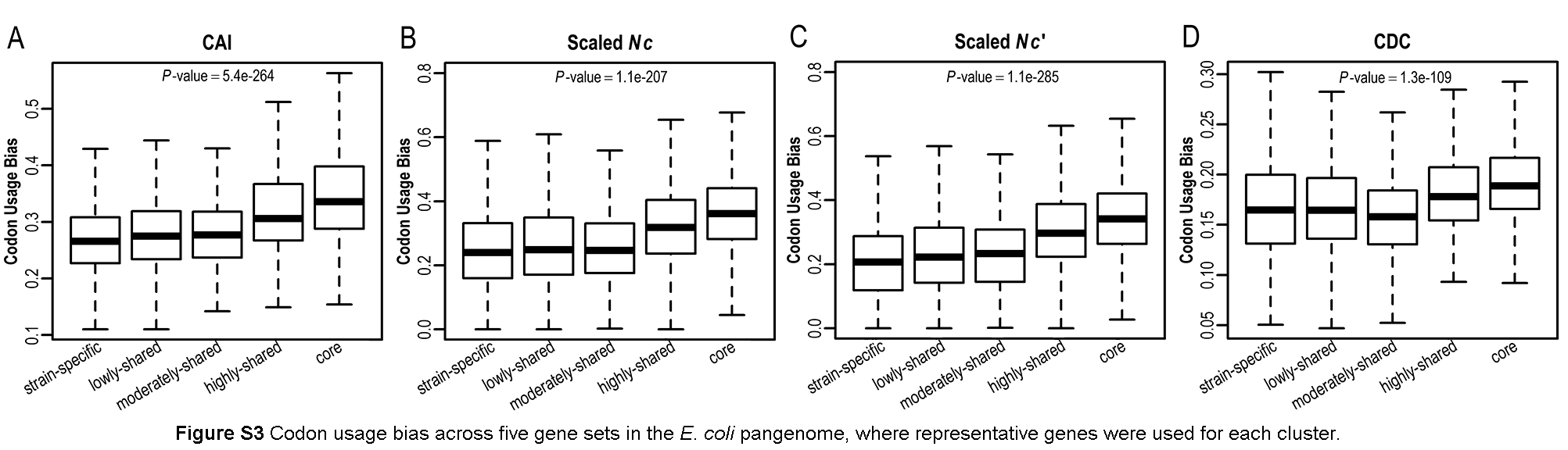

Supplement: Supplementary file 10 [file Image3.TIF]

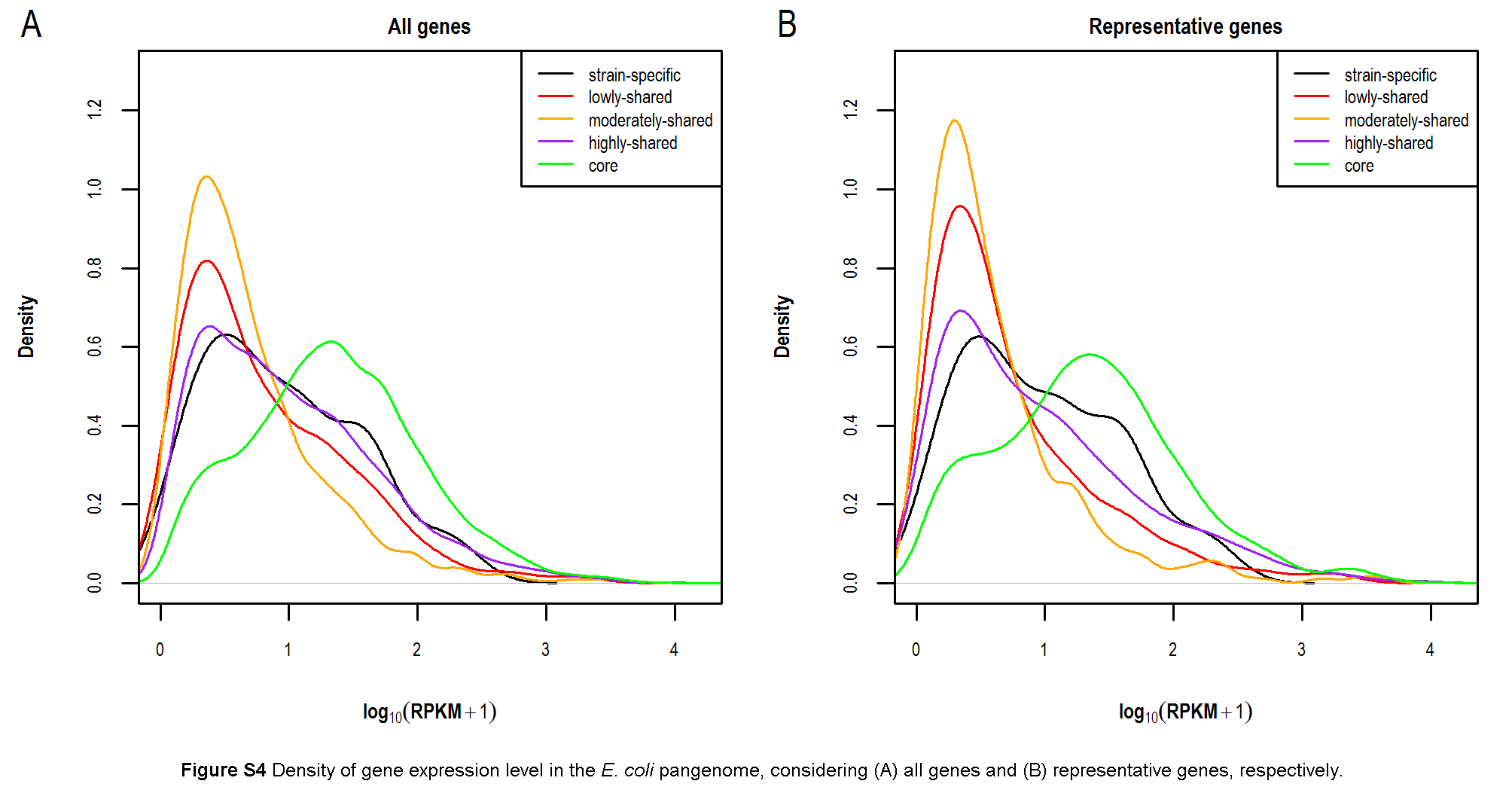

Supplement: Supplementary file 11 [file Image4.TIF]

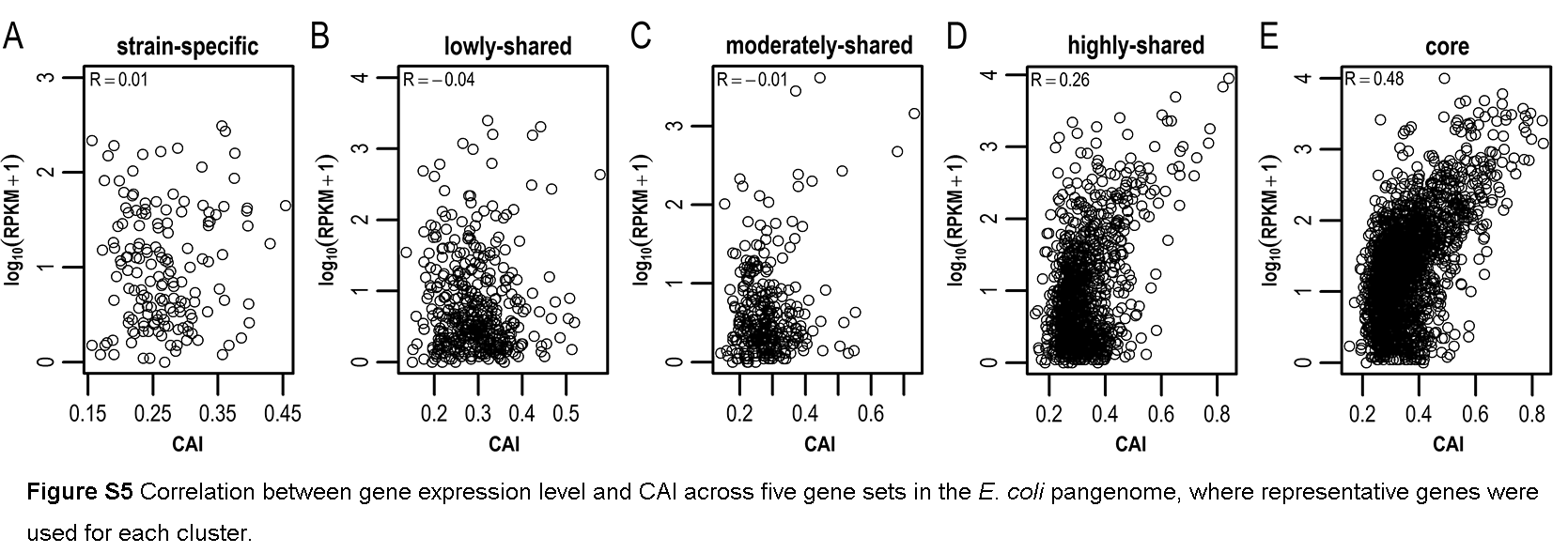

Supplement: Supplementary file 12 [file Image5.TIF]

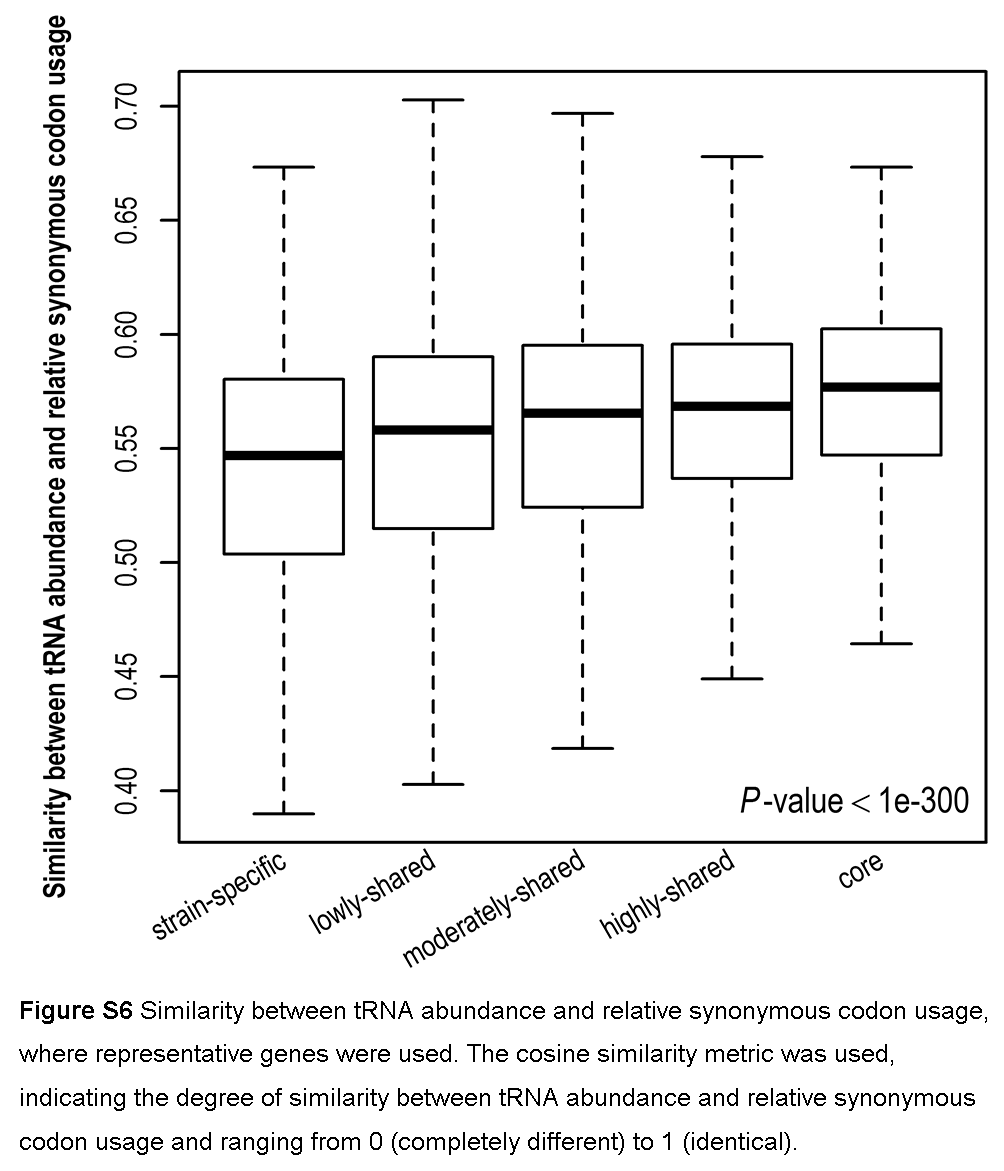

Supplement: Supplementary file 13 [file Image6.TIF]

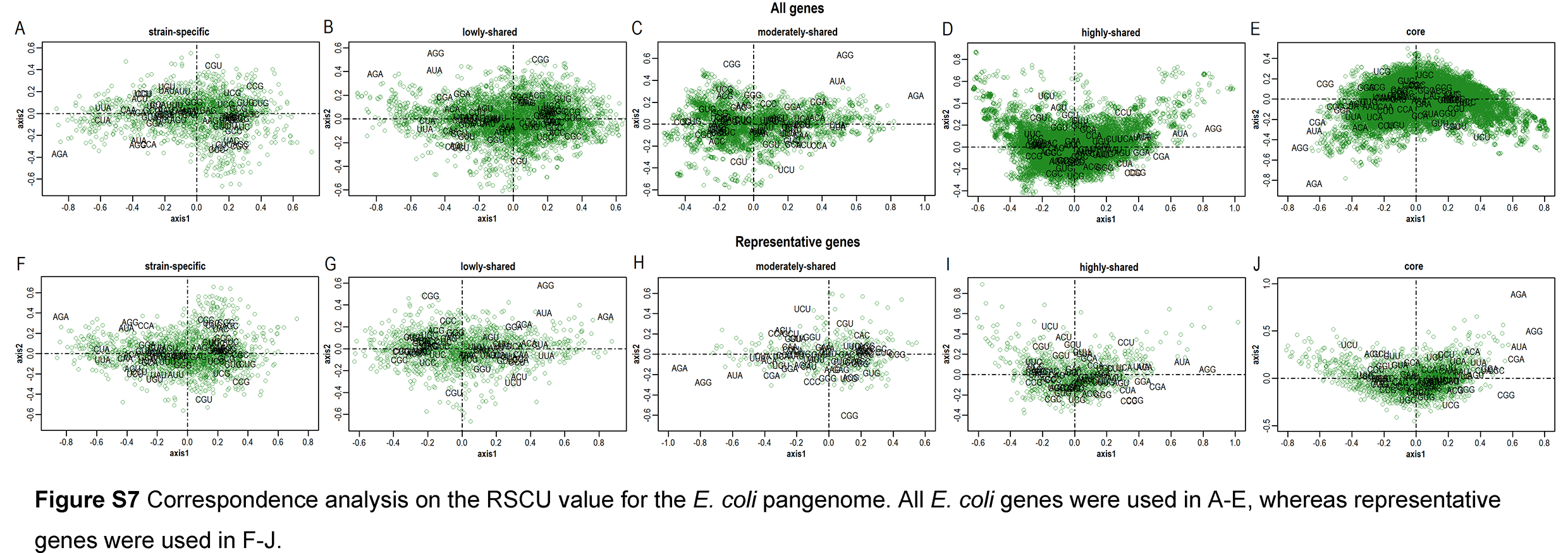

Supplement: Supplementary file 14 [file Image7.tif]

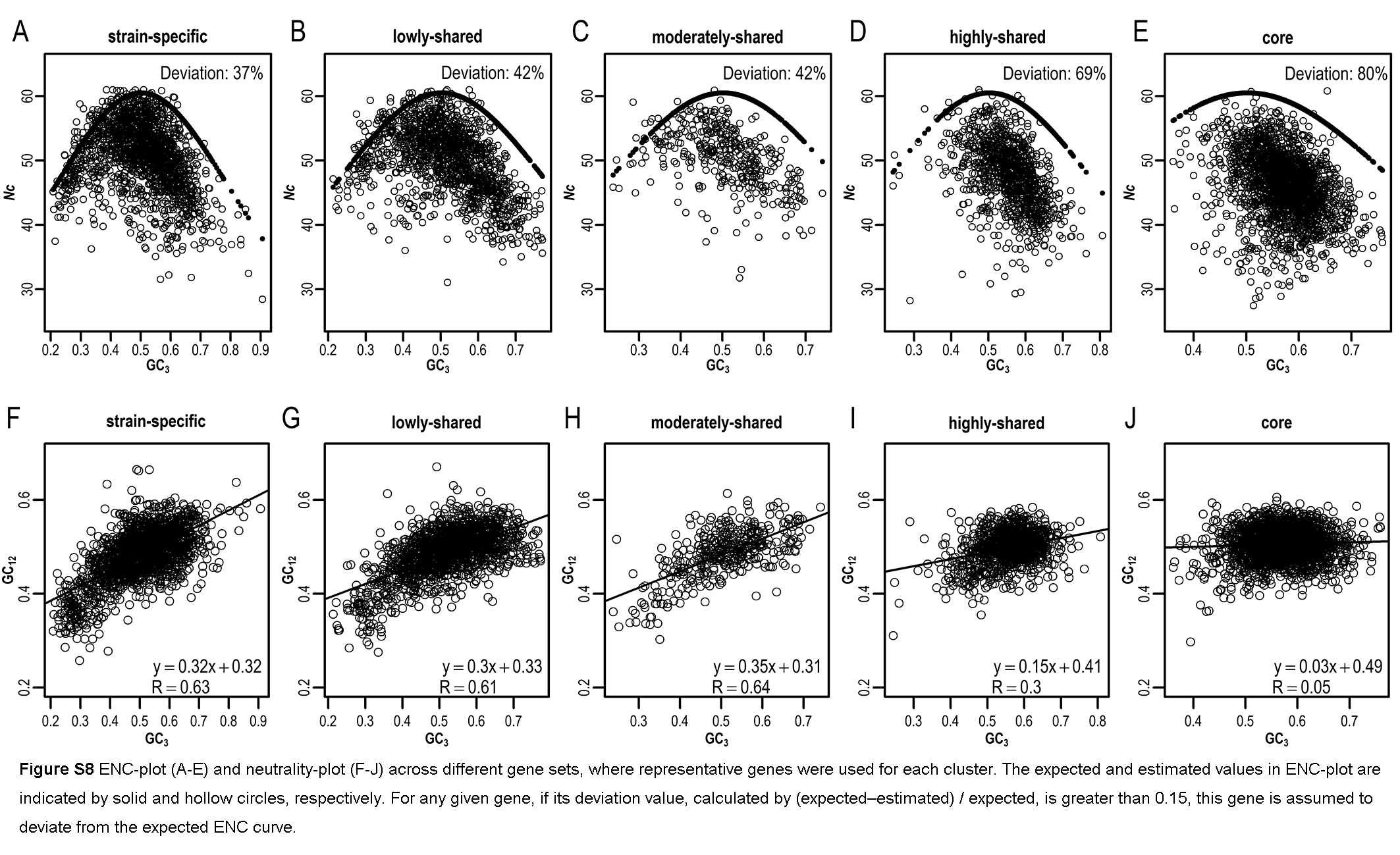

Supplement: Supplementary file 15 [file Image8.TIF]
